# Mental Disorders among Homeless People in Western Countries

**DOI:** 10.1371/journal.pmed.0050237

**Published:** 2008-12-02

**Authors:** Helen Herrman

## Abstract

Helen Herrman discusses the implications of a new systematic review and meta-analysis of the prevalence of mental disorders in homeless people in Western countries.

Much has been written in the scientific literature and the popular press about mental disorders among homeless people, and about its converse—homelessness among people living with mental disorders. Yet despite community concern about homelessness and mental illness, we have limited understanding in many places of the genesis of the problem, and limited evaluation of useful ways to tackle it [1–4]. There is little discussion of national surveillance of homelessness and mental illness and a lack of reliable estimates of its extent.

## Systematic Review and Meta-Analysis of Prevalence Rates

In a new systematic review and meta-analysis published in this issue of *PLoS Medicine*, Seena Fazel and colleagues note that “more reliable estimates of the prevalence of serious mental disorders in the homeless should help inform public policy and development of psychiatric services, particularly in urban centres” [5]. They find that despite large variation in findings across studies and countries, homeless people in Western countries are substantially more likely than the general population to have alcohol and drug dependence and psychotic disorders. The prevalence rates of other mental disorders are also likely to be higher than in the general population. The authors note that the presence of serious mental disorders in homeless people contributes to increased rates of death from suicide, drug abuse, and other causes, and is a risk factor for violent victimisation, criminality, and longer periods of homelessness.

The main mental health problems for homeless people in Fazel and colleagues' analyses were alcohol and drug dependence, with random effects pooled prevalence estimates of 38% and 24%, respectively. The proportionate excess for psychosis, however, was greater than for the other mental disorders, with a random effects pooled prevalence estimate of 13%. This finding is consistent with the attention given to psychosis in homeless people by mental health planners and community rights activists. Homeless people with psychotic disorders typically have multiple comorbidities, including substance use disorders, physical illnesses, and disabilities, and often experience a poorly coordinated response to complex needs [6].

**Linked Research Article**

This Perspective discusses the following new study published in *PLoS Medicine*:

Fazel S, Khosla V, Doll H, Geddes J (2008) The prevalence of mental disorders among the homeless in Western countries: Systematic review and meta-regression analysis. PLoS Med 5(12): e225. doi:10.1371/journal.pmed.0050225

Seena Fazel and colleagues show, through a systematic review and meta-regression analysis, that homeless people in Western countries have a higher prevalence of alcohol and drug dependence and mental disorders.

Fazel and colleagues draw these conclusions from the studies available over the last 40 years in a set of Western countries. It is a stark finding that despite all that has been written about mental illness among homeless people, and all the speculation about the origin of the problem, the authors deemed only 29 studies relevant to their analysis. One of the reasons for this relatively small body of work is the absence of clear definitions of homelessness and mental disorders in many publications.

## What Is Homelessness?

Homelessness is defined in various ways, but studies need to include a clear definition, and as implied by Fazel and colleagues, one that is locally relevant. The term can include people living in marginal accommodation, as well as roofless people. Some studies include the former, while in others the definition includes only those sleeping in designated shelters or public spaces. The features of a definition of homelessness that are useful in any locality, however, are the lack of appropriate housing and the social marginalisation of the individual [4]. Also important to policy and planning in any locality is the recognition that people may be transiently, episodically, or chronically homeless [7], and that entry to or exit from the homeless state is usually part of a process rather than a single jump.

## Evidence and Speculation about Homelessness and Mental Disorders

The substantial heterogeneity between the studies included in Fazel and colleagues' systematic review is expected and was analysed carefully by the authors. This analysis supports the authors' contentions that the findings are useful for drawing broad conclusions about the need for policy and practice change in a range of countries, while emphasising that service planning should not use the summary estimates given in the paper but should rely instead on local studies of morbidity to quantify needs. Several technical factors are likely to contribute to variation in the findings, as the authors outline. In comparing information across studies and countries, the definitions of homelessness, sample selection, and diagnostic criteria all need to be considered, as well as other features of the studies such as participation rates. Some of the variation can also be ascribed to local factors such as housing and social service provision. Changes in the affordability of alcohol may contribute to increasing rates of alcohol dependence over time, as may variation in policing and community attitudes.

Beyond local surveys of morbidity, countries or regions need relevant information collected in a consistent way over time. Qualitative studies are also needed that provide information from intimate observation or that record the views and comments of people who are homeless and living with mental disorders [8]. The mental health service, accommodation, and support needs of homeless people require careful study in each country. Policy and practice changes need to be evaluated for their effects on prevalence and on the social inclusion of individual people at risk of or experiencing episodes of homelessness.

## Mental Disorders among Homeless People

The solutions needed are likely to vary considerably by type of disorder, despite commonalities in some of the serious consequences such as victimisation, criminality, suicide, and death from other causes. Homelessness among people living with psychotic disorders, for instance, is often linked to deinstitutionalisation in Western countries, although the analysis of the apparent failure of community care does not support a causal role [2]. An analysis of needs and barriers to meeting needs can help set research and policy direction [6]. Overcoming the mistaken views that disability is inevitable and that nothing really helps, and that people with psychotic disorders have different needs and wants from others in the community, is the first step.

Demonstration programmes in the United States concluded that homeless adults with severe mental illnesses, often thought to be beyond the reach of existing outreach services, are willing to accept psychiatric treatment and can remain in community-based housing with appropriate support. Housing stability, appropriate psychiatric treatment, and increased income can lead to an improved quality of life [9–11]. Accessible mental health services for homeless people, including outreach services, can encourage helpful service contact [3,12], and homeless mentally ill people in Australian settings, for instance, will use accessible primary health care [13].

## Implications for Policy and Planning

The rate of mental disorders among homeless people is useful information for advocacy and for monitoring policy and practice change in a community. However, it does not in itself give a good indication of service needs, and contact with psychiatric services does not necessarily imply that health needs are being met [13]. To answer these questions requires the assessment of disabilities, including self-reports [3,14,15]. Homeless people living with mental disorders by and large require social support, housing, and economic security, in addition to mental health services, to alter their life circumstances [16]. The effectiveness and critical ingredients of inter-agency partnerships that address the complex needs associated with homelessness in people with psychosis require investigation [6].
